# In Vivo Confocal Microscopy of the Human Cornea in the Assessment of Peripheral Neuropathy and Systemic Diseases

**DOI:** 10.1155/2015/951081

**Published:** 2015-12-07

**Authors:** Ellen F. Wang, Stuti L. Misra, Dipika V. Patel

**Affiliations:** Department of Ophthalmology, New Zealand National Eye Centre, Faculty of Medical and Health Sciences, The University of Auckland, Auckland 1142, New Zealand

## Abstract

In vivo confocal microscopy (IVCM) of the living human cornea offers the ability to perform repeated imaging without tissue damage. Studies using corneal IVCM have led to significant contributions to scientific and clinical knowledge of the living cornea in health and pathological states. Recently the application of corneal IVCM beyond ophthalmology to wider clinical and research fields has been demonstrated. Abnormalities of the corneal subbasal nerve plexus have been associated with many forms of peripheral neuropathy and Langerhans cells correlate with systemic inflammatory states. There is a rapidly growing evidence base investigating the use of corneal IVCM in many systemic conditions and a well-established evidence base for IVCM imaging of the corneal subbasal plexus in diabetic peripheral neuropathy. This paper reviews the potential use of corneal IVCM in general clinical practice as a noninvasive method of assessing peripheral neuropathies, monitoring inflammatory states and clinical therapeutic response.

## 1. Introduction

The cornea is the most densely innervated tissue in the body [1]. The cranial nerves, with the exception of the optic nerve, are considered to be a part of the peripheral nervous system, with all the sensory nerves being derived from neural crest cells during embryology [2]. Therefore corneal nerves, arising from the ophthalmic branch of the trigeminal nerve, are considered a part of the peripheral nervous system [2]. The nerves enter the midstroma to track anteriorly from the periphery into the centre in a radial pattern, losing their myelin sheath within 1 mm of the limbus to aid corneal transparency [1, 3, 4]. Corneal nerves are comprised of large, myelinated A*δ* fibres that run parallel to Bowman's layer and small, unmyelinated C-fibres that run parallel to Bowman's layer for a short course and then penetrate the epithelium to terminate in invaginations within the superficial cells [1, 3, 4].

**Figure 1:**
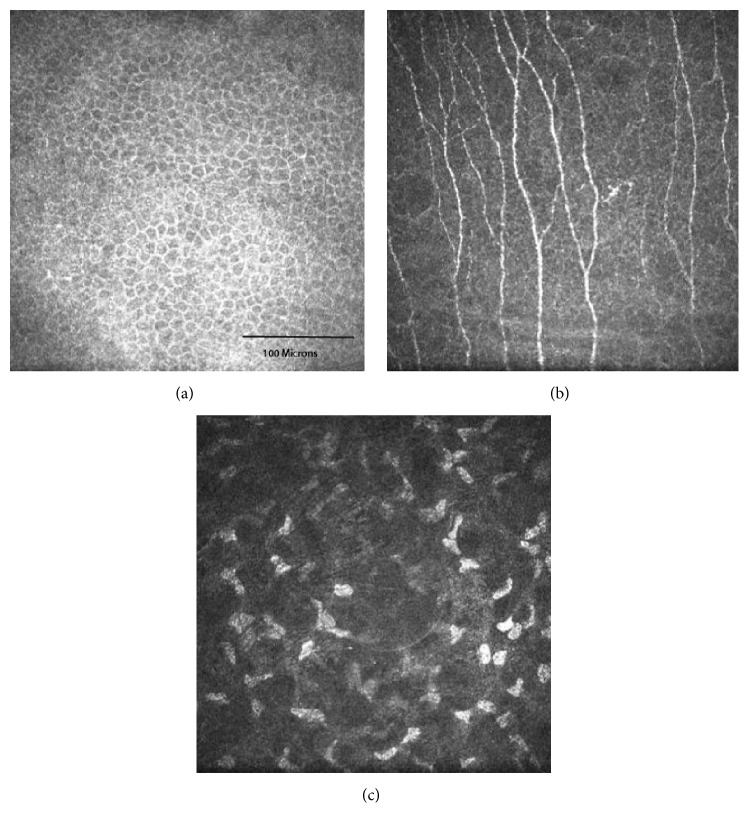
In vivo confocal microscopy images showing the normal corneal basal epithelium (a), central corneal subbasal plexus (b), and stroma (c) from a healthy 24-year-old female.

Living human corneal nerves can be imaged noninvasively using in vivo confocal microscopy (IVCM) (Figure 1) [3–6]. Three different modes of IVCM have been developed: laser scanning confocal microscopy, slit-scanning confocal microscopy, and tandem scanning confocal microscopy [3–5]. These modes vary in terms of light emission, magnification, contrast, and resolution but all offer the ability to repeatedly examine the same cornea without tissue damage.

IVCM has been widely utilized in clinical practice in corneal and ocular surface imaging. It is used as an aid in the diagnosis of* Acanthamoeba* keratitis, in the assessment of keratoconus, dry eyes, and contact lens wear [5]. Repeated examination of the same cornea allows for detection of changes that are used to monitor response to therapy and recovery after corneal surgery [5]. As well as enabling imaging of corneal pathology, IVCM is increasingly being investigated for its potential to evaluate systemic disease. Studies using this technique have demonstrated corneal subbasal nerve changes that correlate with peripheral neuropathies such as diabetic peripheral neuropathy, idiopathic small fibre neuropathy, Fabry disease, and HIV associated peripheral neuropathy (Table 1) [7–13]. IVCM has also been used to evaluate the ocular effects of neurodegenerative conditions, rheumatological conditions (Table 2), genetic diseases, and chemotherapy.

The current review discusses the contribution of IVCM in the diagnosis and assessment of these systemic diseases and its ability to overcome limitations in current clinical methods of assessing these conditions. This review also aims to explore IVCM's potential use in clinical diagnosis, monitoring disease progression, and therapeutic response in current standard clinical practice and in clinical trials of novel treatments, thereby contributing to clinical knowledge and informed clinical decision making.

## 2. Diabetes

Diabetes is a disease that is of growing global significance, now affecting over 366 million people worldwide [14]. It affects multiple organ systems with its microvascular complications causing the well-known clinical triad of nephropathy, retinopathy, and neuropathy, with diabetic peripheral neuropathy being the most common [15]. These complications lead to decreased quality of life for patients and an increasing burden on global healthcare systems and can result in debilitating long term sequelae such as foot ulceration, amputations, blindness, and renal failure.

Current methods of detecting diabetic peripheral neuropathy include taking a medical history and an examination that involves a clinical peripheral nervous examination, bioesthesiometry, and invasive nerve conduction studies [16–18]. These methods often detect diabetic peripheral neuropathy when the neuropathy becomes well established and have limited sensitivity for detecting early diabetic peripheral neuropathy [7, 16, 18, 19]. While tight glycaemic control is a known key factor in the clinical management of diabetes, it limits the progression of neuropathy in type 1 diabetes, but not type 2 [1]. There is no pharmacological method of preventing or reversing diabetic peripheral neuropathy [1, 16, 20]. Current methods of detecting peripheral neuropathy have several disadvantages including their subjective and variable nature and while electrophysiology and nerve biopsies are reliable, they are expensive and invasive [1, 15, 21].

**Figure 2:**
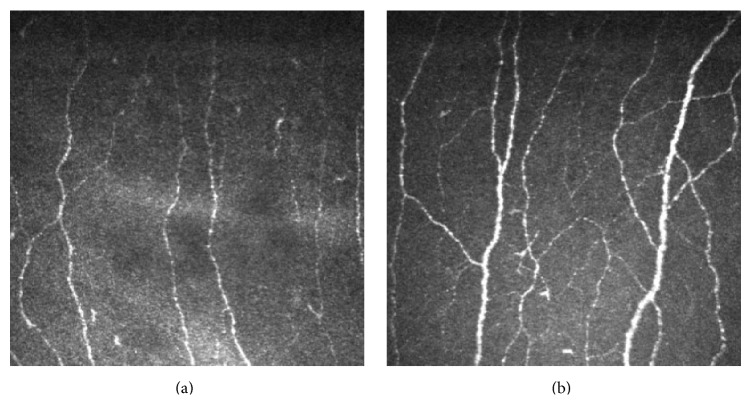
In vivo confocal microscopy images showing the corneal subbasal plexus of a 33-year-old female with a 14-year history of type 1 diabetes (a) and a healthy 32-year-old healthy female (frame size represents 400 *μ*m × 400 *μ*m).

IVCM is a rapid, noninvasive, and accurate method that enables quantitative analysis of the corneal subbasal nerve plexus and could potentially provide a surrogate marker for diabetic peripheral neuropathy [1, 14, 19]. The total length of corneal nerves per unit area is reported to be the most reproducible measure of corneal subbasal nerve density in patients with diabetes [22]. Studies have also shown that patients with type 1 or type 2 diabetes exhibit a marked reduction in subbasal nerve density compared to healthy corneas (Figure 2, Table 3) [8, 14]. Importantly, 50% of patients with diabetes who had no clinical signs of neuropathy were shown to have abnormal corneal subbasal nerve plexus changes, demonstrating corneal changes precede clinically detected peripheral nerve changes [19].

Corneal subbasal nerve density correlates with clinical and electrophysiological assessment of the severity of diabetic peripheral neuropathy [14, 16, 23]. Decreased subbasal nerve density is associated with symptoms of peripheral neuropathy and decreased intraepidermal nerve density [16, 23]. Interestingly, these changes in corneal nerve density precede any clinical signs or symptoms of neuropathy, retinopathy, and microalbuminuria and are also seen in patients with impaired glucose tolerance, without meeting clinical criteria for type 2 diabetes mellitus [7, 8, 14, 16, 24, 25].

IVCM has also been used to assess the recovery of type 1 diabetic patients that undergo pancreas and kidney transplantation [26, 27]. In these studies the focus of IVCM imaging was to detect a therapeutic response to transplantation [26, 28]. Patients receiving simultaneous pancreas and kidney transplantation suffer from severe diabetic consequences, including neuropathy [26–28]. Introducing functioning islet *β*-cells via pancreas transplantation into patients with type 1 diabetes reverses some of the end organ damage caused and although slow to improve diabetic nephropathy it reportedly improves diabetic neuropathy within one year of receiving the transplant [27]. Regeneration of the intraepidermal nerve fibre layer is an important outcome measure of transplant success and can only be assessed by performing skin biopsies. The correlation of corneal subbasal plexus density with intraepidermal nerve fibre density suggests the potential use of IVCM for monitoring therapeutic response [26, 28]. This could open the door to further studies in this application to other systemic conditions, including the conditions mentioned in this review.

## 3. Neurodegenerative Diseases

### 3.1. Parkinson's Disease and Progressive Supranuclear Palsy

Parkinson's disease (PD) and progressive supranuclear palsy (PSP) are neurodegenerative movement disorders that have similar clinical presentations [29]. Both PD and PSP patients are noted to have ocular surface disease in the form of dry eye syndrome, exacerbated by the decreased blink rate associated with these movement disorders [30]. This is thought to occur due to a denervation of the cornea, leading to reduced corneal sensitivity and reduced blink rate, resulting in asymptomatic surface disease [30]. A study of a small group of patients with a diagnosis of either PD (4 patients) or PSP (7 patients) reported significantly reduced corneal sensitivity and blink rate compared to healthy age-matched controls. However, there was no difference in corneal subbasal nerve density between the three groups despite reduced corneal sensitivity [30]. This observation suggests that the ocular effects of Parkinson's disease and PSP may be due to neural dysfunction rather than denervation but require further investigation due to the small numbers of patients studied [30].

Peripheral neuropathy associated with PD is common and reported to affect 38–55% of patients and the risk of developing peripheral neuropathy in PD may be greater in those treated with levodopa [31]. A study investigating the relationship between corneal nerve density and Parkinson's related peripheral neuropathy examined 25 patients with Parkinson's disease and 25 healthy control subjects. All underwent corneal sensitivity testing with a Cochet-Bonnet aesthesiometer and IVCM. Patients with Parkinson's disease were reported to have significantly decreased corneal sensation, lower corneal nerve density, and greater nerve tortuosity compared with control subjects. The parameters measured were also noted to be related to exposure to dopaminergic medication, inferring the potential use of IVCM to monitor patients receiving dopaminergic medication although the nature of this relationship was not specified [32].

### 3.2. Amyotrophic Lateral Sclerosis

Amyotrophic lateral sclerosis (ALS) or Lou Gehrig's disease is the most common degenerative disease of motor neurons [33]. Progressive degeneration of upper and lower motor neurons in the brain and spinal cord leads to decreased coordination, speech or voice changes, and cognitive changes that eventually result in muscle atrophy and spasticity, and ultimately death [33]. Most cases are sporadic and the exact aetiology is unknown although several gene mutations have been identified in cases of both familial and sporadic ALS [33]. While ALS largely is a disease of motor neurones, the sensory nervous system is also known to be involved, causing a decrease in intraepidermal nerve fibre density [34–36].

Subclinical sensory neuron involvement has been demonstrated with pathological evidence on sural nerve biopsies taken from ALS patients [35, 37]. A study of 8 sporadic ALS patients demonstrated that although none of the patients had any signs or symptoms of sensory neuropathy, there was a clear reduction in corneal subbasal nerve density compared with age-matched healthy control subjects [37]. Disease burden was assessed using the ALS Severity Score and the revised ALS Functional Rating Scale [37]. Severity was assessed with 4 outcome measures based on function: speech, swallowing, lower extremities, and upper extremities with speech and swallowing comprising the “bulbar subscore” and the lower and upper extremities comprising the “spinal subscore” [38]. Interestingly, corneal nerve damage correlated with bulbar disability scores but not with spinal disability [37]. In this case series, IVCM has revealed the possibility that ALS is associated with a small fibre neuropathy and further IVCM studies may bring us closer to understanding the mechanisms behind the poorly understood aetiology of ALS.

### 3.3. Idiopathic Small Fibre Neuropathy

Idiopathic small fibre neuropathy (ISFN) is a subset of small fibre neuropathy that occurs due to damage of the small unmyelinated peripheral nerves, known as C-fibres. The neuropathy is sensory in nature but symptoms are highly variable in location and severity. Typically the symptoms begin in the extremities and while impaired glucose tolerance has been a suggested risk factor, it is not present in all cases and symptoms do not seem to be related to nerve damage. Idiopathic small fibre neuropathy is therefore more difficult to diagnose than other neuropathies [39], particularly since nerve conduction studies evaluate large myelinated fibres and do not test the small unmyelinated fibres. Quantitative sensory testing is subjective and unreliable, meaning clinicians are increasingly turning to skin biopsies for a conclusive diagnosis.

A pioneering IVCM study of 24 patients with idiopathic small fibre neuropathy showed a significant decrease in corneal subbasal nerve density and increased nerve fibre tortuosity [39]. Corneal subbasal nerve changes were not correlated with impaired glucose tolerance, body mass index (BMI), lipid levels, or blood pressure [39]. This is an interesting observation given that all the patients in this study had significant neuropathy symptoms but normal electrophysiology and quantitative sensory testing [39]. Another small case series of three patients with ISFN also demonstrated significantly decreased corneal subbasal nerve density compared with normal controls, correlating with intraepidermal nerve fibre density from skin punch biopsies [40]. Whilst these studies are too small to draw concrete conclusions, they point to the fact that the mechanism behind ISFN is still poorly understood and thus far only associations with metabolic risk factors can be made without any causal relationships.

### 3.4. Charcot-Marie-Tooth Disease

Charcot-Marie-Tooth (CMT) disease is the most common hereditary motor and sensory neuropathy that causes progressive loss of muscle tissue and sensation [41, 42]. CMT is categorised into subtypes based on the gene mutation that is present with the most common subtype being type 1A, accounting for 70–80% of all cases [42]. The diagnosis is based on history and genetic testing, clinical examination, and nerve conduction testing [41, 42]. Although disease progression is attributed to demyelination and axonal degeneration of large myelinated fibres, there is no correlation between motor or sensory nerve conduction testing and neurological disability scores in CMT1A [41, 43].

CMT is associated with a small fibre neuropathy (C-fibres) but this has been difficult to assess and quantify. Electrophysiology does not test the small unmyelinated fibres involved in this neuropathy and therefore patients with CMT are traditionally investigated with subjective quantitative sensory testing and with invasive skin or sural nerve biopsies [42]. In a study 12 patients with CMT1A were recruited with 12 age-matched healthy subjects and all underwent a detailed neurological examination including bioesthesiometry, nerve conduction studies and symptoms of neuropathy, corneal sensitivity measured using a noncontact corneal aesthesiometer, and IVCM [42]. Patients with CMT1A were reported to have significantly decreased corneal subbasal nerve density, correlating strongly with symptoms of painful neuropathy and reduced nerve conduction testing scores [42].

IVCM has an emerging role in enabling clinicians to quantify the small fibre pathology in CMT1A. IVCM offers a rapid, noninvasive evaluation of CMT1A, offering an advantage over quantitative sensory testing and biopsies.

## 4. Rheumatology

### 4.1. Systemic Lupus Erythematosus

Systemic lupus erythematosus (SLE) is a chronic inflammatory autoimmune condition of varying severity that can affect any organ system. It is of unknown aetiology and its pathogenesis involves the production of autoantibodies due to loss of T-cell regulatory ability and results in complement activation that triggers the inflammatory cascade [44]. It has a variety of ocular manifestations including optic neuropathy, retinal vasculitis, and keratoconjunctivitis sicca with keratoconjunctivitis sicca being the most common [44].

**Figure 3:**
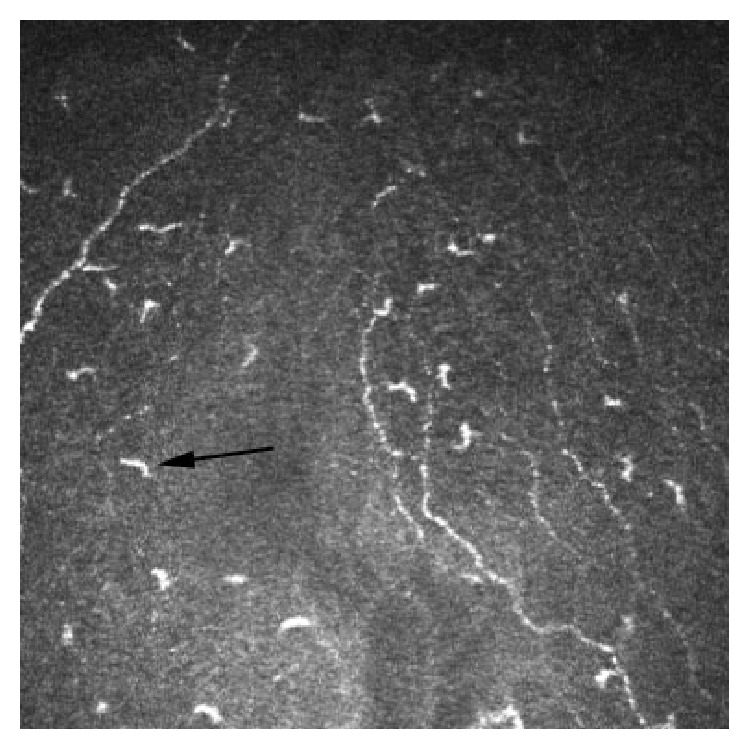
In vivo confocal microscopy image at the level of Bowman's layer showing Langerhans cells (arrow) (frame size represents 400 *μ*m × 400 *μ*m).

SLE related keratoconjunctivitis sicca demonstrates a marked increase in the density of Langerhans cells within the central cornea, with over half of these cells exhibiting dendritic features, as imaged by IVCM (Figure 3) [45]. Langerhans cells are a subset of the population of antigen presenting dendritic cells within the cornea, mainly located in the epithelium of the peripheral cornea [46]. They activate T-cells as part of the corneal immune system [46]. In pathological states Langerhans cells mature, form dendritic processes, and migrate from the periphery into the central cornea [46].

Interestingly, IVCM has been used to assess a case of bilateral deep keratitis associated with SLE induced iridocyclitis [47]. The patient presented with decreased visual acuity during a flare-up of SLE and examination revealed deep stromal opacities spread throughout the central and peripheral cornea. IVCM was used to further assess the nature of these opacities and subsequently demonstrated deposition of refringent crystals within the corneal stroma [47]. These deposits mirror the deposition of immune complexes in systemic tissues in patients with SLE. The crystalline deposits are located in the deep stroma and are believed to occur as a result of dilation of perilimbal vessels during inflammatory episodes [47–49].

While rare, corneal manifestations can be the initial presentation of SLE and in these cases IVCM of corneal deposits may contribute towards diagnosis. However, further studies are needed to clarify its role in diagnosis and to investigate the potential of using IVCM to monitor the progression of deep keratitis as a biomarker for disease severity [47]. IVCM may also aid in the assessment of keratoconjunctivitis sicca, the most common ocular manifestation of SLE [44]. Further studies of IVCM imagining of Langerhans cells in the central cornea in SLE could help establish a relationship between this and disease progression and systemic inflammation, potentially providing another biomarker for clinical monitoring of SLE.

### 4.2. Ankylosing Spondylitis

Ankylosing spondylitis (AS) is a seronegative chronic inflammatory condition primarily of the axial skeleton, with variable involvement of the peripheral skeleton [50]. AS typically affects young males with the most common presenting complaint being lower back pain due to sacroiliac joint inflammation [50]. Progression of the disease leads to bone formation in the spine which results in fusion of the joints [51].

The association between AS and uveitis is well known [52]. AS is also associated with corneal changes; the most common is keratoconjunctivitis sicca due to secondary Sjögren's syndrome, occurring in 10% of patients [50].

As in SLE, Langerhans cells play an important role in AS related keratoconjunctivitis sicca. There is also increasing evidence to suggest that Langerhans cells in the cornea play an important role in the immunoregulatory processes [50]. Studies investigating the role of these cells in the cornea have noted that Langerhans cells in the cornea tend to be activated [44, 46, 53]. Langerhans cells then migrate from their usual location in the peripheral cornea, into the central cornea during an inflammatory state [44, 46, 50, 53].

An increased number and activation of Langerhans cells were not associated with articular disease symptom severity but correlated with patients with higher systemic inflammatory levels [i.e., higher serum C-reactive protein (CRP) and erythrocyte sedimentation rate (ESR)] [50]. Due to the small numbers in this study (24 patients) associations between Langerhans cell density and HLA B27 could not be made [50]. Patients with an active systemic inflammatory disease state (elevated serum CRP and ESR) had significantly decreased tear production compared with healthy control subjects and AS patients with low systemic inflammation [50]. This suggests that corneal changes are associated with systemic inflammation rather than dry eye and poses the possibility that IVCM of Langerhans cells, much like SLE, can be used to monitor disease progression and therapeutic response [44].

### 4.3. Rheumatoid Arthritis

Rheumatoid arthritis (RA) is a chronic, inflammatory disorder affecting the joints, leading to progressive deformation and loss of function [54]. The inflammation affects the joint capsule and cartilage, causing fibrosis, stiffness, and pain. Systemically RA can cause diffuse inflammation of the pleura, pericardium, and ocular surfaces [54]. Ocular manifestations of RA include scleritis, keratitis, and keratoconjunctivitis, with sicca symptoms being the most common [53]. IVCM allows for direct visualisation of the ocular tissues, especially the Langerhans cells that play a key role in regulation of the corneal immune response [53].

The maturation process by which Langerhans cells develop dendrites and migrate to the central cornea is induced by inflammatory cytokines including IL-1*α*, IL-6, IL-8, IL-12, and TNF-*α* [53]. As with systemic lupus erythematosus and ankylosing spondylitis, IVCM of the central cornea in 52 RA patients and 24 age-matched controls revealed an increase in dendritic, activated Langerhans cells in the central cornea of those with RA [53]. Langerhans cell density at the central cornea was 68.15 ± 71.27 cells/mm^2^ in RA patients and 23.85 ± 33.81 cells/mm^2^ in healthy control subjects [53]. This remains a significant increase even after patients with overlapping Sjögren's syndrome and eye symptoms were excluded [53]. IVCM allows for an integrated, whole body approach to assessment of rheumatoid arthritis where disease severity can be measured in terms of not only systemic inflammatory markers, but also clinical ocular signs of keratoconjunctivitis sicca and Langerhans cell density.

### 4.4. Sjögren's Syndrome

Sjögren's syndrome is a chronic autoimmune disorder in which the exocrine glands are affected by inflammation leading to xerostomia and keratoconjunctivitis sicca. The syndrome can be primary or may occur secondary to another connective tissue disorder [54]. Previously, keratoconjunctivitis sicca associated with Sjögren's syndrome was thought to be due to a deficiency in the secretion of the aqueous component of the tear film, but now it is recognised to be a complex interaction between aqueous deficiency, lacrimal gland inflammation, and evaporative dry eye [55].

Sjögren's disease shows an increased density of dendritic Langerhans cells in the central cornea, as imaged by IVCM. This increase is observed in several other systemic diseases, previously mentioned in this review [46, 50, 53]. Langerhans cell density could possibly be a useful marker of disease status in Sjögren's syndrome.

Another biomarker investigated in Sjögren's disease is conjunctival goblet cell density and meibomian gland density [55, 56]. Both primary and secondary Sjögren's patients demonstrated lower meibomian gland density. However, Sjögren's syndrome shows less acinar dilation, lower secretion reflectivity, and decreased gland opening diameters in contrast to patients with meibomian gland disease [55]. A decrease in conjunctival goblet cell density is reported to be an accurate marker of ocular surface disease [56]. This is due to the role of the goblet cells in secreting the mucin needed for tear film stability and a reduction in the number of goblet cells is associated with ocular surface disease [56]. Traditionally assessment of goblet cells is made by impression cytology [56]. IVCM enables imaging of goblet cells in vivo and a significant decrease in goblet cell density has been demonstrated in Sjögren's syndrome patients noninvasively [56]. However it is of note that goblet cell density measured by IVCM was higher than the density measured by impression cytology [56]. Examination of goblet cell density allows for not only diagnosis of Sjögren's syndrome, but also evaluation of the efficacy of therapeutic interventions and monitoring of disease progression [56].

## 5. Genetic Diseases: Fabry Disease

Fabry disease is a rare genetic, X-linked lysosomal storage disease characterised by a deficiency or absence of the alpha-galactosidase A enzyme. This deficiency results in the accumulation of a metabolic by-product known as globotriaosylceramide (Gb3), a glycosphingolipid. The accumulation of glycosphingolipid in vasculature, tissues, and end organs produced a clinical myriad of symptoms that can include renal failure, hypertension, cardiomyopathy, neuropathy, anhidrosis, and angiokeratomas [57]. Presentation of Fabry disease varies and includes cardiomyopathies, renal impairment, unexplained pain throughout the whole body or localised in the extremities, within the abdomen and thoracic cavity, and gastrointestinal and cerebrovascular symptoms [58]. The variability in clinical presentation makes Fabry disease an often elusive diagnosis. A review of Fabry disease demonstrated that the delay in diagnosis can be as long as 10 years, a significant delay in a potentially life-threatening disease [57]. Corneal verticillata is a characteristic ocular sign of Fabry disease, occurring in 88% of all female and 95% of all male patients and are thought to occur due to glycosphingolipid deposition within the cornea at the level of Bowman's layer [57].

Corneal verticillata can be caused by a variety of agents, usually long term therapy with a wide variety of medications (e.g., amiodarone), or can occur secondary to environmental exposure to silica dust [57]. Verticillata is hyperreflective intracellular inclusions, and their appearance on IVCM as an irregular surface on Bowman's layer helps to differentiate Fabry disease from other causes such as amiodarone-induced keratopathy [12, 58]. IVCM also reveals generalised deposition of a reflective substance throughout the corneal stroma [12]. In addition to IVCM's diagnostic merit, it has also been demonstrated to be useful in monitoring the regression of the hyperreflective inclusions in patients being treated with enzyme replacement therapy [10].

Fabry disease is associated with a small fibre neuropathy, a key factor in the diagnosis and monitoring of disease progression [18]. Electrophysiology and quantitative sensory testing have been employed for monitoring purposes but are known to be less accurate than the more invasive techniques of sural nerve or skin biopsies [18]. Symptom severity, electrophysiology, and quantitative sensory testing (QST) all correlated significantly with a decrease in subbasal corneal nerve density on IVCM, making IVCM a potentially accurate and noninvasive monitoring tool in this disease [18].

## 6. Immunology

### 6.1. HIV

Human immunodeficiency virus (HIV) associated peripheral neuropathy is the most common neurological effect of the virus and affects most HIV patients to varying degrees [59]. As with small fibre neuropathies of other causes, HIV peripheral neuropathy is associated with a significant decrease in intraepidermal nerve fibre density [60]. HIV neuropathy can be assessed through reported symptoms, quantitative sensory testing, and skin biopsies of intraepidermal nerve fibre density, each with their own limitations. Patient reported symptoms are subjective, and often symptoms of mild neuropathy are not reported [60, 61]. Quantitative sensory testing of vibration, warm and cool sensation are able to detect individuals with the greatest degree of peripheral nerve dysfunction when correlated with invasive skin punch biopsies [61]. While these methods are able to identify those with HIV associated neuropathy, they are not sensitive enough to detect mild neuropathy [61].

A key study investigating simian immunodeficiency virus (SIV), the simian variant of HIV, has shown that SIV leads to significant corneal subbasal nerve loss in Macaque monkeys [62]. The study involved* in vitro* immunolabeling of corneal tissue from Macaque monkeys infected with SIV followed by manual and automated analysis of nerve density [62]. Corneal nerve density was directly correlated with epidermal nerve fibre length, measurement of the degree of RNA viral replication, and cellular immune activation in the trigeminal ganglia [62]. In addition to these correlations, corneal nerve fibre density was reported to be lower in the group with faster progression of neuropathy, signalling the potential to use IVCM as a monitoring tool for the progression of disease [62].

This study has been heralded as a cornerstone development in the study of HIV neuropathy. If this is also demonstrated in human patients, IVCM could provide effective diagnosis of HIV neuropathy and be a valuable clinical tool in monitoring the progression of HIV neuropathy [62].

### 6.2. Peripheral Autoimmune Neuropathy

Peripheral autoimmune neuropathy is a group of syndromes involving an acquired, chronic inflammatory process that results in peripheral nerve damage. Several different immune pathways have been implicated but the process is still poorly understood. Chronic inflammatory demyelinating polyneuropathy (CIDP) is one such syndrome and while it mainly affects large myelinated nerves, skin biopsies of CIDP patients have also demonstrated small nerve fibre involvement [63].

IVCM demonstrated significantly reduced corneal subbasal nerve density in CIDP patients [63]. Despite this, subbasal nerve density did not correlate with nerve conduction studies, trigeminal somatosensory evoked potentials, or any other clinical measurement. This lack of correlation points towards the multifocal nerve fibre involvement hypothesis in a poorly understood condition [63].

Another case noted normal subbasal nerves but significantly thickened and tortuous stromal nerves that returned to normal appearances after treatment with rituximab and corticosteroids [11]. These corneal nerve changes correlated with clinical symptoms and nerve conduction studies [11]. The observation of decreased subbasal nerve density in CIDP could contribute to overall medical knowledge and understanding of the underlying pathogenesis of this condition. The application of IVCM to this condition requires further investigation. Larger prospective studies are needed to explore the relationship between corneal subbasal nerve density and the pathogenesis and progression of CIDP. Its ability to simultaneously assess small fibre damage and the immune response in dendritic, maturing Langerhans cells makes IVCM an attractive potential tool in the diagnosis and monitoring of CIDP.

## 7. Chemotherapy Induced Peripheral Neuropathy

Chemotherapy with platinum based agents such as oxaliplatin is considered the standard of care in treatment of gastrointestinal cancers [64]. Unfortunately the drug may cause sensory neuropathy in up to 80% of patients, a neuropathy sufficiently severe to limit the dose of chemotherapy delivered or terminate treatment altogether [64]. Recently, corneal IVCM has been shown to be a sensitive clinical tool in early diabetic peripheral neuropathy and may be clinically used to diagnose and monitor progression of neuropathy. Therefore, it may be possible to use corneal IVCM as a surrogate or even prognostic marker for chemotherapy induced peripheral neuropathy.

**Figure 4:**
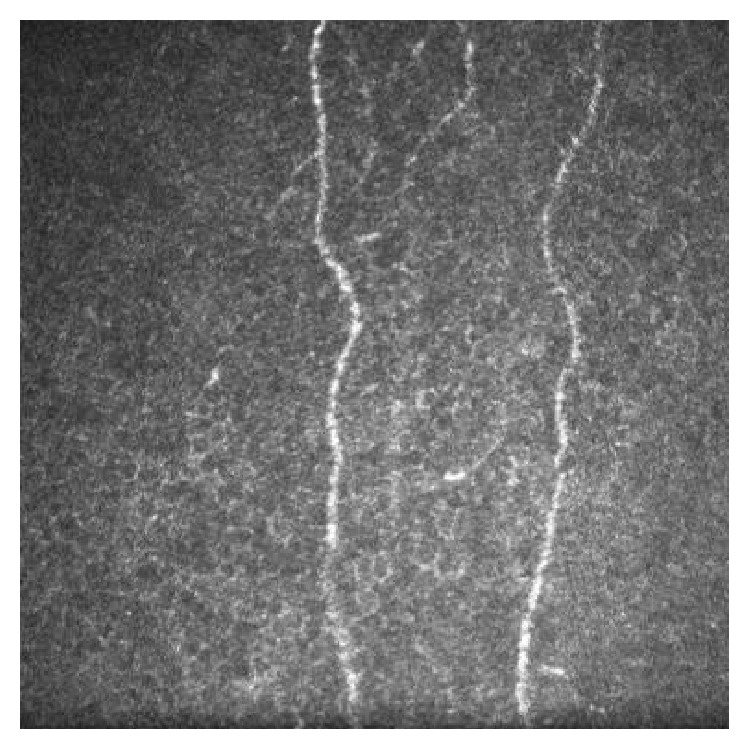
In vivo confocal microscopy image of the central corneal subbasal plexus of a 72-year-old male who completed nine courses of oxaliplatin chemotherapy, showing a low subbasal nerve density (frame size represents 400 *μ*m × 400 *μ*m).

In the largest case series in the literature, fifteen patients receiving oxaliplatin chemotherapy for colorectal adenocarcinoma were assessed prior to commencement of chemotherapy, after four cycles in those undergoing more than four cycles, and again after completion of chemotherapy [9]. Out of the fifteen oxaliplatin patients, ten had worsened subjective neuropathy symptoms at the end of the study and eight had clinically detected signs of peripheral neuropathy. IVCM revealed corneal subbasal nerve plexus abnormalities in ten patients, with a decreased nerve density and increased tortuosity (Figure 4). Seven of these patients had worsened symptoms of neuropathy and abnormal neurophysiology; the remaining three had worsened symptoms and normal neurophysiology. Of the five patients with stable symptom scores, four had IVCM changes. IVCM changes were present after four cycles and persisted until completion [9].

This case series highlighted the potential role IVCM may play in oxaliplatin-induced peripheral neuropathy and how it may be used to identify at-risk patients. Future studies investigating the use of IVCM in oxaliplatin-induced peripheral neuropathy are needed to fully assess the correlation between corneal subbasal nerve density and peripheral neuropathy. Further assessment may reveal corneal IVCM to be a cost-effective, rapid screening tool for detecting or predicting oxaliplatin-induced peripheral neuropathy and monitoring its development during chemotherapy treatment.

## 8. Conclusion

IVCM allows for repeated, noninvasive, direct visualisation of these nerves, enabling detection of damage, making it a powerful clinical and research tool [4, 5]. Recently there is increasing interest in applying this technique to the assessment of systemic conditions and peripheral neuropathies with hopes that its advantages will provide a rapid, cost-effective method of assessing and managing patients.

Peripheral neuropathies are currently evaluated using several methods such as electrophysiology, assessment of neurological disability via various validated questionnaires, and quantitative sensory testing [15, 21, 24, 65]. These clinical parameters have several limitations when assessed in a clinical setting. Questionnaires are subjective, and while electrophysiology and quantitative sensory testing methods are objective, they are often not sensitive enough to detect the early stages of neuropathy. Only the gold standard of an invasive nerve or skin biopsy will permit clinicians to directly examine nerve fibre damage. IVCM is able to overcome these limitations and most importantly allows for repeated examinations without causing tissue damage.

There is growing evidence regarding the use of IVCM in patients with diabetes and correlating corneal nerve density with peripheral neuropathy. However, only a handful of clinical studies have investigated the use IVCM in other systemic conditions, and most of the studies involved are small case series. While providing interesting results that suggest IVCM could be clinically useful in these conditions, further research is needed to fully explore its diagnostic and monitoring potential.

There is an increasing body of research investigating the use of IVCM of the cornea in patients with systemic diseases. However, while being promising much of the research in this field involves small numbers of patients. Despite showing statistically significant correlations, further work is needed to evaluate its potential use in the diagnosis and management of systemic disease.

## Figures and Tables

**Table 1:** Corneal subbasal nerve densities on in vivo confocal microscopy in a range of systemic diseases.

	Corneal subbasal nerve density in patients (mm/mm^2^)	Corneal subbasal nerve density in controls (mm/mm^2^)
Diabetes type 1 [14]	20.6 ± 1.5	27.7 ± 1.1
Diabetes type 2 [24]	4.3 ± 1.5	13.5 ± 0.3
Parkinson's disease [32]	15.0 ± 8.0	13.5 ± 5.0
Progressive supranuclear palsy [32]	15.0 ± 6.0	13.5 ± 5.0
Amyotrophic lateral sclerosis [36]	1.8 ± 0.4	2.3 ± 0.4
Idiopathic small fibre neuropathy [38]	4.4 ± 0.6	9.3 ± 0.6
Charcot-Marie-Tooth type 1A [41]	15.8 ± 1.5	26.7 ± 1.3
Chronic inflammatory demyelinating polyneuropathy [62]	18.1 ± 3.4	23.5 ± 3.6
Chemotherapy induced peripheral neuropathy [9]	6.8 ± 2.4	10.8 ± 3.8

**Table 2:** Langerhans cell density on in vivo confocal microscopy in the central and peripheral cornea in rheumatological conditions.

	Central Langerhans cell density in patients (cell/mm^2^)	Central Langerhans cell density in controls (cell/mm^2^)	Peripheral Langerhans cell density in patients (cell/mm^2^)	Peripheral Langerhans cell density in patients (cell/mm^2^)
Systemic lupus erythematosus [45]	43.08 ± 48.67	20.57 ± 21.04	124.78 ± 165.39	78.00 ± 39.51
^*∗*^Ankylosing spondylitis [49]	75.50 (51.18–112.6)	14.50 (0.00–35.10)	131.0 (80.33–168.4)	65.50 (46.75–88.00)
Rheumatoid arthritis [52]	68.15 ± 71.27	23.85 ± 33.81	126.8 ± 104.6	69.29 ± 33.26

^*∗*^Figures for ankylosing spondylitis are expressed as median with interquartile range and others as a mean with standard deviation.

**Table 3:** Corneal subbasal nerve densities on in vivo confocal microscopy in diabetes mellitus.

	Corneal subbasal nerve density in patients (mm/mm^2^)	Corneal subbasal nerve density in controls (mm/mm^2^)
Petropoulos et al. [14], type 1 diabetes		
No retinopathy	20.6 ± 1.5	27.7 ± 1.1
Retinopathy	17.4 ± 0.9	27.7 ± 1.1
No microalbuminuria	19.9 ± 1.7	27.7 ± 1.1
Microalbuminuria	14.3 ± 1.4	27.7 ± 1.1
Misra et al. [19], type 1 diabetes	11.0 ± 3.8	21.17 ± 4.2
Malik et al. [24], type 2 diabetes		
Mild	10.8 ± 0.9	13.5 ± 0.3
Moderate	7.5 ± 1.1	13.5 ± 0.3
Severe	4.3 ± 1.5	13.5 ± 0.3
Tavakoli et al. [23], type 2 diabetes		
Mild	5.48 ± 0.45	11.21 ± 0.88
Moderate	3.01 ± 0.39	11.21 ± 0.88
Severe	2.99 ± 0.34	11.21 ± 0.88
